# ‘They really want to find some kind of box they can put you in’: how young people with severe mental health problems and complex needs talk about their past treatment in traditional child and adolescent mental health services - a critical discourse analysis

**DOI:** 10.3389/fpsyt.2026.1759231

**Published:** 2026-04-14

**Authors:** Marthe Johansen, Ottar Ness, Hanne Kilen Stuen, Eva Brekke, Anne Landheim

**Affiliations:** 1 Department of Health and Nursing Sciences, University of Inland Norway, Elverum, Norway; 2 Department of Education and Lifelong Learning, Norwegian University of Science and Technology, Trondheim, Norway; 3 Research Center for Substance Use Disorders and Mental Illness, Innlandet Hospital Trust, Brumunddal, Norway

**Keywords:** child and adolescent mental health services, Fairclough critical discourse analysis, neoliberal governance, person-centred care, severe mental health problems, young people, youth flexible assertive community treatment

## Abstract

Challenges in child and adolescent mental health services (CAMHS), including complex and fragmented services, are well documented, particularly to young people with severe mental health problems and complex needs. Research on young people’s experiences of CAMHS is scarce. Especially studies on young people who have dropped out of CAMHS and/or transitioned to alternative services are lacking. This article addresses the following research question: Which discourses can be identified when young people with severe mental health problems and complex needs enrolled in Youth Flexible Assertive Community Treatment (Youth Flexible ACT) articulate their past treatment in CAMHS, and contrast it with their current treatment? We conducted a qualitative study using semi-structured interviews with 12 participants aged 15 to 19, recruited via convenience sampling from three Youth Flexible ACT teams in Norway. The interview data were analysed using a Fairclough-inspired critical discourse analysis (CDA), where discourse is understood as a particular way of understanding and talking about the world, and language is seen as a social practice rather than an individual activity. We identified three dominant discourses when the young people talked about conventional services/CAMHS in Norway: a biomedical, a paternalistic and a neoliberal. The findings suggest that treatment in CAMHS is shaped by a market-oriented understanding of mental health (neoliberal discourse), a medical model that reduces complex problems to medical terms (biomedical discourse), and a lack of user participation (paternalistic discourse). The findings indicate a gap between what young people see as helpful, the aims outlined in guidelines and the young participants’ descriptions of CAMHS. The lack of user involvement and the unequal power balance need to be actively addressed with concrete practices that support more equal participation. The findings highlight the need for more differentiated treatment pathways for this group of young people, enabling timely, tailored support and reducing the risk of ineffective initial interventions. Our findings indicate that the framework in CAMHS is poorly suited to the needs of this group. In contrast, differently organised services, such as Youth Flexible ACT, may offer an alternative by providing a coherent framework for holistic, integrated and person-centred care.

## 1 Introduction

Global estimates from large-scale meta-analysis, show that nearly half of all mental disorders begin before the age of 18, with a median age of 14,5 years across diagnostic categories (1). Despite this early onset, young people, often report a range of barriers to seeking help with mental health services (2–4).

The current study focuses on young people aged 12–25 years who experience severe mental health problems and complex needs. This population lacks universally accepted definitions (5, 6). In this study, they are defined as young people with significant psychological, functional, and social difficulties who require support from multiple services. The emphasis is placed less on diagnostic categories and more on challenges in daily functioning. Barriers and facilitators to help-seeking among young people are multifaceted. A recent review (4) identified stigma as the most prominent barrier, consistent with previous findings (3, 7), followed by negative attitudes and beliefs about mental health services and professionals. Structural barriers, such as cost and logistical challenges were reported, though less frequently (3). The most reported facilitators were strong and trusted relationships with gatekeepers (e.g. teachers, parents, GPs, and other health professionals), as well as previous positive experiences with help-seeking.

Challenges in child and adolescent mental health services have been well documented (2, 8, 9), including complexity and fragmentation of services (10) and an inability to meet young people’s developmental needs (2). There is a need to develop mental health services that meet these challenges (2, 9, 11). To achieve this The World Health Organisation (WHO) (12) and experts advocate that an integrated, holistic and systemic understanding should underpin service provision (12–14). Many government documents and reports in Norway point out similar shortcomings in mental health care for young people. Examples include a lack of individual tailoring, flexibility, service capacity, and user involvement (15–17). This applies in particular to those with severe mental health problems and complex needs, and with concurrent substance abuse problems (16–18).

In Norway, mental health services are responsible for the assessment and treatment of young people (primarily to age 18) with mental health problems. These services are mainly provided through child and adolescent mental health services (CAMHS). which are organized as outpatient and inpatient clinics. Young people are referred to CAMHS regardless of the complexity of their problems. CAMHS operates within a framework defined by organisational, legal and bureaucratic factors, including requirements for service user involvement, efficiency and evidence-based knowledge (19, 20). In CAMHS, treatment is time-limited and evidence-based, emphasising assessment and diagnosis and followed mainly by talk therapy, and when appropriate medication. Interventions are intended to be tailored to the young person’s individual needs. For young people with severe mental health problems and complex needs (age range, 12–25 years) who do not benefit from conventional services, Youth Flexible assertive community treatment (Youth Flexible ACT) teams were established in Norway in 2020 (21). As of October 2025, approximately 20 teams were active. Youth Flexible ACT is an adapted variant of the adult. Flexible ACT model (22). The teams are multidisciplinary, with members from different professional backgrounds contributing on an equal basis. They operate as assertive outreach teams for children and young people aged 12–25 years who have severe mental health problems and complex needs. This includes individuals with possible problematic substance use and a presumed need for long-term and complex service provision. In Norway, the teams are organised as a binding collaboration between primary and specialist mental health care services, integrating two care levels in one team (21). The core principles of the Youth Flexible ACT model include flexibility, assertive outreach and a multidisciplinary team approach. These principles aim to promote participation in the local community, strengthen family and systemic work, and support recovery and user involvement. The model further seeks to ensure continuous, holistic and integrated follow-up, and to use evidence-based methods and practices (23).

International studies on young people’s experiences with CAMHS indicate that they have varying experiences with these services. Common themes revolve around relational aspects, like being listened to, taken seriously and meaningfully involved in decisions. Several challenges have been identified, including poor continuity, a lack of trust in staff, a lack of information about mental health services and the treatment process, and a lack of involvement in decisions (24–27). There are scant qualitative studies in child and adolescent psychiatry (28) specifically exploring the experiences with CAMHS of young people with severe mental health problems (29, 30). Davidson et al. (31) found that vulnerable young people often felt that they were not listened to or taken seriously, and they experienced CAMHS staff to be inaccessible with long waits for appointments or responses. In another study by Kapur et al. (32), young people hearing voices expressed a need for less stigmatising and more holistic care. By contrast, young people’s experiences with Youth Flexible ACT indicate a high level of overall satisfacition (33). They reported trusting and collaborative relationships with staff. They also emphasised the importance of the services being organised in a holistic, flexible and accessible manner (33).

Standard mental health services are not suitable for all young people; those with severe mental health problems and complex needs often receive services that are insufficiently equipped to address their multiple and complex needs (29, 34). It is therefore important to engage with these youth to gain more insight into their experiences. There is a need for insights from young people who have dropped out of conventional services/CAMHS (27), especially those with severe mental health problems who have experienced ineffective treatment (29). Understanding their experiences can inform the development of more differentiated and tailored services.

Young people’s experiences and mental health services are part of a broader context shaped by dominant interpretive frameworks (discourses). This article employs critical discourse analysis, viewing young people’s experiences through the social, cultural and institutional contexts they inhabit (35). Discourse analysis places findings within a comprehensive interpretive framework, opening new perspectives (36). A discourse perspective examines how people and their actions are shaped by the language they use to describe the world (35), which includes the analysis of youth language. This approach may be vital in studies of children and young people as they engage in complex interactions with the systems, relationships and services around them. Discourse analysis aligns with the perspective that young people in treatment contexts should be studied across multiple dimensions, due to the complex interactions involved. Using critical discourse analysis, the study examines how young people receiving treatment from Youth Flexible ACT articulate their experiences. Further to identify discourses and shed light on how power, social norms, perspectives, and institutional understandings are reflected in the language utilized by the young people when describing their experiences with CAMHS. Finally, the study explores the implications of these discursive patterns for the developmentof tailored treatment services.

The aim of this study is to critically analyse how young people in a Youth Flexible ACT context construct and describe their past treatment in child and adolescent mental health services (CAMHS). The analysis examines how these constructions are informed by social, cultural and linguistic contexts, including comparisons with their current Youth Flexible ACT treatment. This article addresses the following research question: Which discourses can be identified when young people with severe mental health problems and complex needs enrolled in Youth Flexible assertive community treatment articulate their past treatment in CAMHS, and contrast it with their current treatment?

### 1.1 Perspectives and paradigms

In order to explore the discourses highlighted when young people talk about their previous experiences of CAMHS, we will initially describe different perspectives or paradigms that can form a basis for understanding mental health problems and their treatment: 1) Mental health care can be considered within a *neoliberal framework* (37), in which patients are seen as consumers who are responsible for their own health and who demand the right type of health care, and where health services are viewed as being governed by rational control mechanisms such as efficiency and cost-benefit (38, 39). Studies have found that a neoliberal discourse dominates mental health care (40, 41). 2) The *biomedical model* is considered to occupy a hegemonic position in international psychiatry, with its roots far back in history. The diagnostic system is grounded in this paradigm and the current preoccupation with neurobiological psychiatry can be seen as a rebiologisation or remedicalisation of mental health care (42). In this paradigm, mental health problems are seen as illnesses to be cured, which originate in biochemical or genetic abnormalities (43, 44), rather than involving an aim to improve people’s health. 3) *Paternalism* in mental health care refers to an asymmetrical power relationship in which professionals decide on the methods and theories to be used, and where patients are dependent on the professional’s choices and on how much user involvement is permitted (41). 4) The *biopsychosocial model* includes a variety of perspectives to explain mental illness. It is based on the premise that biological, psychological and social factors all play a role in illness, where none of these factors is inherently superior to the others (45, 46). 5) *A recovery approach* to mental health care is now recommended internationally by the World Health Organisation (WHO) (47). Many understandings of recovery-oriented practices exist, but we refer to frameworks where Recovery means focusing on strengths rather than illness and quality of life instead of just symptom reduction, and the person is seen in the context of the system in which she or he lives (48, 49). The limited but growing literature on personal recovery in children and young people with mental health problems (50, 51) finds that it involves elements such as agency, participation, youth-friendly language and a family focus, and that young people have a less consistent view of recovery than adults (52). 6) *Service user participation* is an important element in the recovery approach and is stipulated as a legal right (53).

## 2 Materials and methods

### 2.1 Design

This is a qualitative study inspired by critical discourse analysis with a social constructionist framework. The study is based on the assumptions that reality is socially constructed and that the world is dependent on social, historical and cultural processes (54). The research design is exploratory and interpretive. The exploratory element involved the interview guide, which was developed partly inductively and in an exploratory manner, and the data collection using semi-structured interviews, where we wished to explore participants’ experiences. The interpretive element was our use of critical discourse analysis, which is both a theory and a method of analysis. In this article, we refer to the young people’s *language* use rather than what they experience in order to emphasise that we analyse the written language in the interview transcripts, not the participants’ individual thoughts and intentions. The way in which participants talk about the treatment is considered to reflect social discourses and practices in the mental health service being analysed.

#### 2.1.1 Fairclough’s critical discourse analysis

In this study, we used Fairclough’s critical discourse analysis (CDA) as inspiration for the theory and methods used. According to Fairclough’s CDA, discourse is described as a particular way of understanding and talking about the world, where language is seen as a social practice rather than an individual activity. The role of language and text in the production and reproduction of power and domination is central to this method. Fairclough explores relationships in which power is exercised in a way that has negative consequences for certain social groups, aiming to promote more equitable social relations (55–57). By examining how young people talk about their experiences of traditional mental health services, underlying assumptions where some worldviews are taken for granted and others are marginalised can be illuminated (58).

This study draws on semi-structured interviews with young people and explores how they discursively construct and make sense of their encounters with mental health services. The interviews are treated as situated accounts produced in interaction and shaped by broader social and institutional discourses, rather than as direct reports of ‘true’ experiences in a realist sense. Accordingly, the analysis does not claim representativeness. Instead, we offer an analytically grounded account of the discursive resources and subject positions that are mobilised in the material, enabling conceptual insights that may be transferable to similar contexts.

### 2.2 Setting

The setting was three Youth Flexible ACT teams, which started in the spring/summer of 2020. The three teams were multidisciplinary, comprising psychologists, nurses and peer specialists. Two teams also included a social worker, a doctor and a mercantile. Several staff worked part time in the Youth Flexible ACT team while also being employed in other municipal or specialist services. In May 2022, 55 young people were receiving services from the three teams.

### 2.3 Recruitment and participants

Convenience sampling was used with the aim of recruiting participants who could provide experiences of treatment from a Youth Flexible ACT team (59). The inclusion criteria were participants who 1) spoke Norwegian fluently enough to have no need of an interpreter and 2) currently received support from a Youth Flexible ACT team that had lasted at least six months. Staff from the three Youth Flexible ACT teams, preferably those who knew the young person best, provided information about the study. We wrote a study information sheet for the participants, which the staff handed out. These were young people with various difficulties that might affect their ability to participate in research, such as little initiative and poor attendance., The following steps in the recruitment process were taken to facilitate participation: 1) Those who wanted to participate signed a written consent and gave it to the staff or the interviewer. The staff coordinated the recruitment process and interview appointments were made in consultation with the first author. 2) Appointments were made by the staff or by phone between the interviewer and the young person. 3) We made repeated appointments with several of them to facilitate their participation (33).

Twelve young people who, at the time of the study, were receiving Youth Flexible ACT and had prior experience of CAMHS were included in this study. We did not collect detailed information about participants’ previous experiences with CAMHS, as the interviews were not primarily focused on this theme. However, most of the young people said they had experiences with outpatient treatment in CAMHS, one said she had experienced inpatient and outpatient treatment, and several had received repeated episodes of care in CAMHS. Three boys and nine girls (mean age 16 years, age range 15–19 years) participated. All participants and the parents of the one participant under 16 years signed an informed consent. All reported having psychological problems and/or diagnoses, including depression, psychotic problems, childhood trauma, anxiety, high-functioning autism, post-traumatic stress disorder, eating disorder and personality disorder. All participants reported school problems ranging from minor difficulties in adapting to being absent from school for the last 2–3 years. One participant reported problematic substance use. Some described family conflicts as the reason for receiving support from the Youth Flexible ACT team. Most participants reported past or present experiences of multiple psychological, functional and social problems in their lives (33).

### 2.4 Data collection

Data were collected in January and February 2022 by the first author, a clinical psychologist who had previously worked with children and young people. The interviews took place at a location of the participants’ choice. Most interviews were conducted at the Youth Flexible ACT teams’ offices, and some in a school or a park. Nine interviews were conducted face-to-face, while three were conducted over the phone. The participants spoke with the interviewer alone. The length of the interviews ranged from 25 to 60 minutes, but most lasted about 50 minutes. The interviews were audio-recorded, except for one that the participant did not want to be recorded, but notes from this interview were included in the analysis. The audio recordings and the transcribed material were stored in pseudonymised form on a secure server at Innlandet Hospital. The transcripts were not checked by participants. Participants were compensated with a gift card worth NOK 500 (about USD 47).

The interview guide was designed to encourage participants to speak freely about how they experienced the follow-up and treatment they were currently receiving in Youth Flexible ACT, without directing them explicitly towards any previous services.

A semi-structured interview guide with open-ended questions and prompts was designed focusing primarily on the young people’s experiences with Youth Flexible ACT, and containing six main topics and associated subtopics. The main topics were: 1. how the young people experience the follow-up and treatment they receive from the Youth Flexible ACT team, 2. whether Youth Flexible ACT is different from previous help or treatment they have received, 3. whether they feel they are allowed to take part in deciding what to work on in Youth Flexble ACT, 4. how they experience the team’s collaboration with other services, 5. whether they feel their life has changed after receiving follow-up from Youth Flexible ACT, and 5. whether they have suggestions for how Youth Flexible ACT can function in a better way. One example of a question that was asked was: ‘How do you experience the follow-up you receive from the Youth Flexible ACT team?’ All were asked the same main questions to ensure consistency in the data collection. In addition, several prompts and questions such as ‘Could you say some more about that?’, ‘Could you give me any examples of that?’, ‘How did it feel?’, and ‘What was it like for you?’ were used to facilitate their ability to respond.

The present article does not address these main topics, except for topic 2 at those points where the participants talked about CAMHS. Rather, it primarily describes the young people’s own accounts of their previous treatment experiences in CAMHS. The semi-structured format, with open-ended questions, enabled participants to construct their own accounts rather than being constrained by predefined categories. During the interviews, many participants spontaneously elaborated on their earlier treatment experiences in CAMHS. These accounts of CAMHS emerged as a recurring and substantial theme and thus formed the empirical basis for the critical discourse analysis presented in this article. The young people’s current treatment in Youth Flexible ACT provides a basis for comparison, and several of the statements draw on a comparison between the two mental health services.

### 2.5 Ethical considerations

This study was approved by the South-Eastern Norway Regional Committee for Medical and Health Research Ethics (REC South-East, 600649). The processing of personal data was assessed by SIKT, the Norwegian Agency for Shared Services in Education and Research (Ref. no. 904802). The study was conducted in accordance with the Declaration of Helsinki. The participants in this study can be considered ‘doubly vulnerable’ according to research ethics guidelines because they are young people in addition to having severe mental health problems (60). Instead of excluding potential participants on the basis of possible severe problems and symptoms, a safety net was established. All participants were asked afterwards how they had experienced the interview. If needed and with a low threshold, the interviewer contacted the team staff to request follow-up. This was done after two of the interviews. All participants were also in treatment from a Youth Flexible ACT team at the time they were interviewed, which ensured that they were cared for by qualified health professionals. They were informed that the interviews were confidential and that participation or non-participation in the project would not affect their treatment. The participants could withdraw from the study at any time without providing a reason (33).

### 2.6 Analysis

To analyse the data, we used Fairclough’s CDA and his original three-dimensional model as our starting point and inspiration for the analysis of the interview transcripts. The advantage of using this analysis is that it provides a structured and simplified method to analyse and deconstruct language (61). Using Fairclough’s three-dimensional framework, we analysed the text based on the three analytical dimensions: 1) as a text, 2) as a discursive practice and 3) as a social practice. Fairclough argues that a text cannot be analysed in isolation, since discourses are dialectical and must be analysed in relation to the meaning of other texts and the social context. In Fairclough’s CDA, discourse is seen as not only *constitutive*, as a form of social practice that reproduces and changes knowledge, identities and social relations, but also *constituted*, as it is shaped by other social practices and structures (51). Which tools the researcher uses from Fairclough’s analysis will depend on the aim of the research and what is to be discussed in the analysis. Fairclough states that the analysis should examine different linguistic aspects (55). The following is a description of the three dimensions in our analysis, including the analytical tools that we decided to use.

#### 2.6.1 Textual analysis

The first and last authors conducted the analysis, using Microsoft Word. We present the steps for the analysis, although these steps were not followed in a strict sequence, but as a back-and-forth movement between the steps. The steps were as follows: (a). We read the transcribed interviews several times to gain an understanding of how the young people’s experiences were textually activated. (b). We read all the sentences and words in the transcripts, where our analytical strategy was a manual search for the verbs, nouns, adjectives and pronouns used by the participants when they talked about CAMHS during the interview. (c). To gain an overview of what the participants talked about and patterns of meaning we categorised the verbs, nouns, adjectives and pronouns semantically, (d). based on this semantic categorisation, we identified possible discourses. (e). We checked the possible discourses against each interview transcript and the data set as a whole to ensure that they were consistently represented in the material. After step (e), we presented the possible discourses to the entire research group, we discussed and reached an agreement on the three prominent discourses in the material. The results of this analysis are summarised in **Table 1 (Appendix 1)**. (f) Further we searched for and analysed the use of grammatical metaphors. (g) and finally we analysed and discussed transitivity and modality. Transitivity is described as a focus on how events and processes are connected to subjects and objects, where the researcher explores how different linguistic forms can have different consequences. In the text, transitivity was seen in statements without an acting subject or with an active object and in whether a passive construction was used instead of an active verb or nominalisation (56). In the analysis of modality, the focus is on the association and confidence the speaker has in relation to her statement. One modality explored in this analysis was the degree of ‘truth’, i.e. the degree of confidence the speaker has in what she says, such as whether the statement implies ‘it’s like that’ or ‘it could be like that’. This is indicated by modal verbs such as can, will, must, should and shall (62).

#### 2.6.2 Discursive practice

Discourse practice entails examining language to identify interconnected discourse patterns that emerge in the interviews, highlighting the roles of intertextuality and interdiscursivity. Inspired by Fairclough, interdiscursivity and intertextuality were analysed. Interdiscursivity means exploring different discourses in a text, and how they draw on each other, are combined or reworked. Intertextuality implies that a text is influenced by history by building on previous texts, and can be implicit or manifest. Manifest intertextuality involves openly building on other texts (55), such as by referring to a diagnostic manual (63).

#### 2.6.3 Social practice

In the third dimension, in line with Fairclough we explored non-discursive social and cultural relations and structures that can create a framework for the discursive practice. Non-discursive elements can be institutional and economic conditions (55).

## 3 Results

The results of the analysis of the participants’ use of verbs, nouns, adjectives and pronouns are summarised in **Table 1 (Appendix 1)**. These findings are further elaborated in the light of Fairclough’s three-dimensional analysis: 1) textual analysis, 2) discursive practice and 3) social practice (55, 56). The results are substantiated by presenting and examining words, wordings and quotes from the interviews. The analysis of the interview transcripts mainly resulted in three discourses: a biomedical, a paternalistic and a neoliberal discourse. The quotations have been anonymized by removing name, age, gender and have been slightly edited for readability and to protect participants’ confidentiality, without altering their substantive meaning. Quotations have been identified by numerical labels (e.g., P1, P2 and so on) to distinguish between participants.

### 3.1 Textual analysis

Using the analytical categories described above, the language in each interview transcript was analysed in relation to the research question.

The analysis shows that young people when talking about CAMHS used words from biomedical terminology, such as diagnosis, psychosis, psychotic, problems, forms, ADHD medication, not normal, different and poor (pitiable).

The participants discussed diagnoses, medication, symptoms, and deviations from normality, all of which are terms from biomedical terminology. They also referred to expressions of symptoms or behaviour, such as becoming violent and paranoid and therefore being referred to CAMHS, but without mentioning the underlying experience or the reason for these problems. These young people generally talked about themselves in medical terms, not in subjective terms.

There were also examples where they used words and phrases suggesting that CAMHS was a facility to control patients and reduce problematic behaviour and symptoms such as: ‘So there were many things that, in a way, affected me, in how I behaved then. I started to stutter, bite my nails and tear at them, and I had very unusual behavior for a small child. I got very violent, so they sent me to CAMHS to control it (P9)’ and ‘[…] so they can give you medicine so you can be less of yourself […] it’s an effective way to get rid of people (P1)’.

The language used suggests that in this biomedical framing, the young people see themselves as having ‘unusual behaviour’ or as problems that need to be controlled, rather than as subjects offered support to meet their needs.

In the textual analysis we also identified paternalistic language such as: ‘doesn’t cooperate, doesn’t listen, strict, fixed system, filling out forms, got a diagnosis, formal, get thrown out, control’. These words reflect a system that provides young people with little agency, and can be described as paternalistic, since the experts (the staff/the system) decide, assess and diagnose.

We found some examples of grammatical metaphors in the transcribed texts. The following quotation shows one of them and is an example of how one of the participants articulated how decisions were made without user involvement in CAMHS:

“One of my friends, she was going to CAMHS, she was thrown out of CAMHS. Here (Youth Flexible ACT), you don’t get thrown out. You don’t get thrown out, if I had just had the period I’ve just been through, I wouldn’t be in CAMHS anymore (P4).”

Being ‘thrown out’ can here be interpreted as a metaphor that suggests rejection and an exercise of power. Rather than saying that treatment had to end, the participant uses language that positions herself and her friend as passive, as people who things are done to, rather than being active participants in decisions about their care.

The participants also used neoliberal language such as: ‘big forms, lots of the same kind of questions, strict, make appointments, fixed appointment, efficient way, easiest solution, mechanical’.

The participants described a system with a fixed framework designed to provide efficiency and cost-benefit to society in line with a neoliberal approach, instead of being adapted to the individual who needs personalised mental health care.

The wording around arena flexibility in the Youth flexible ACT treatment was highlighted as a contrast to the fixed structure in CAMHS, as the following quotation shows:

“The circumstances are calm and you know that they have solutions. It’s not so fixed, everything isn’t set in stone, you know. Do you want us to meet at home, do you want us to meet at the office, do you want to have the meeting somewhere outside, what do you actually want? (P1).”

In this extract, the participant uses the metaphor ‘everything isn’t set in stone’ to characterise the Youth Flexible ACT service. The idea of something being ‘set in stone’ refers to a situation that is rigid and inflexible, which aligns with how CAMHS is described. In contrast, Youth Flexible ACT is portrayed as not having this rigidity. The flexibility is further reinforced by the examples the participant gives of all the choices they are offered regarding where meetings can take place. Within Fairclough’s critical discourse analysis, this can be seen as a vocabulary choice that constructs a discourse of flexibility and user participation, where the young person is seen as a subject with individual meanings and needs.

The participants generally did not use words describing subjective dimensions when talking about CAMHS, such as their thoughts, hopes, wishes and participation. An exception was some of the young people talking about missing their psychologist at CAMHS and that the psychologist understood how they felt.

Transitivity was analysed by examining the roles and responsibilities assigned to the participants. Following Fairclough (57), we explored whether or not responsibilities and roles were attributed to the participants and staff. The following quotation illustrates the use of a construction without an actor: ‘[…] but like I feel in, for example, CAMHS and places like that, there’s a kind of emphasis on how to get rid of your problems quickly, and like more the easiest solution (P1).’ This way of talking that lacks an active subject to get rid of the problems makes it unclear who will perform the actions and thus also with whom the young person could collaborate and negotiate to be involved in treatment decisions.

We did not identify examples of transitivity in the form of nominalisation, but we noted that the participants often spoke in the passive, as seen in this statement: ‘When I was at the clinic, I was sort of given diagnoses (P13)’. The alternative might have been an active verb: ‘They/the psychiatrist gave me diagnoses’. As in the quotation above, which shows the agency assigned to the actors, this passive way of speaking can place greater focus on the action itself, which in this case was diagnosing, than on the person performing the action. The use of passive language can be seen in relation to the participants’ use of the pronoun ‘they’ when referring to CAMHS, which may suggest a division into ‘us’ and ‘them’. ‘They’ is also used to refer to the CAMHS system, indicating that these young people encounter a system that diagnoses them rather than identifiable professionals. Encountering a system makes it unclear who performs actions and thus who is responsible for the actions. In this way the system is constructed as the powerful actor, while the young people are positioned as passive recipients of decisions. This use of language thus constructs identities in which the young people appear as objects of treatment rather than active subjects in their own care.

In terms of the degree of truth modality, the analysis showed that several of the statements had a high degree of truth, resembling ‘it’s like that’ rather than ‘it could be like that’. Quotes that illustrate this are: ‘[…]teenagers don’t get to decide anywhere near as much’, ‘CAMHS don’t have time’ and ‘CAMHS are so strict’. Another participant worded her experience with staff turnover as follows: ‘At CAMHS, it’s kind of something everyone knows, that they are very good at changing staff all the time, and I know very well that I am probably not the first person to say this either (P2)’.

There were also some statements with a lower degree of truth, but these were the exception. The following quotation is an example: ‘And I can imagine that some kids may have quite a problem with making appointments with CAMHS, and it’s much stricter there, because if you don’t turn up for a few appointments, you might not be allowed to go there for much longer (P10)’. By using the modals ‘may’ and ‘might’, this participant was not entirely convinced that what he said about CAMHS was the full truth; this way of speaking leaves some room for nuances.

However, in general, transcribed interview text present a high deegre of certainty in what is presented and few nuances about CAMHS, not by using modal verbs such as ‘must’ or ‘should’, but by expressing ‘that’s the way it is’, as this quote illustrates: ‘It’s often happened [in FACT] that we spend more time than the time they thought I should have, but I could never do that at CAMHS because it is so strict (P7)’.

### 3.2 Discursive practice

Our analysis of the interview transcriptions identified three dominant discursive patterns: a biomedical, a paternalistic and a neoliberal. These three discourses are different but not contradictory; rather, they overlap, which gives the text a low degree of interdiscursivity. According to Fairclough (55), low interdiscursivity indicates that the prevailing order of discourse is maintained, since there are no contradictory discourses in how the participants talk about CAMHS, whereas high interdiscursivity might indicate alternation in the dominant discourse.

Although there is no explicit discursive conflict (high interdiscursivity), several of the participants clearly expressed dissatisfaction with their treatment in CAMHS. This is seen both in their use of language and in their practices, such as skipping appointments or discontinuing their treatment. One said: ‘When I was at CAMHS, I was sort of given diagnoses [ … ] and that’s helped me a lot [ … ]. But I like the way that Youth Flexible ACT is more flexible (P13)’. This statement combines an acknowledgement of the use of diagnosis (biomedical discourse) with a preference for flexibility and participation (person-centred discourse). The description of Youth Flexible ACT as flexible and less ‘illness oriented` suggests a desire for discourses that break with the pathologising logic of CAMHS. This represents interdiscursivity, since different discursive understandings are integrated. Overall, these young people’s resistance to CAMHS and preference for Youth Flexible ACT can be understood as the beginnings of a discursive tension. This tension can potentially challenge and may eventually change the established order of discourse in mental health care for young people. It offers alternatives to the current ways of thinking about what mental health services for this group should encompass.

The participants in this study had in the past had contact with different parts of the health care system for many years, and some had been in treatment for much of their lives. Their use of biomedical language, such as references to diagnosis, medication and assessment, can be understood as expressing intertextuality. This means that previous texts and discourses, such as medical records, treatment plans and conversations with health care professionals, are reused and quoted in their own use of language. Because these institutional discourses are powerful and repeatedly encountered, they become taken-for-granted ways of talking about mental health and provide the participants with ready-made resources for describing themselves and their experiences. For example, their reference to specific diagnoses from diagnostic manuals represents manifest intertextuality. In accordance with Fairclough, the language people use is not randomly chosen but is seen as a form of social practice rather than as an individual activity, and the discourses people draw on are always shaped by previous communicative events (intertextuality) and by the institutional and societal contexts in which they participate (57). For the young people in this study, their often long-term contact with mental health services, have made biomedical, paternalistic and neoliberal ways of talking particularly available to them. This is due to repeated and perhaps regular exposure to this particular terminology, such as the terms used by health care professionals and possibly by their own families, and reinforced through assessments, treatment plans and mental health records. When they talk about CAMHS, they do so through discursive resources that are familiar to them.

In the following sections, we have attempted to present the discourse patterns separately. All of these discourses coexist within the mental health service practice and they clearly overlap, but the main elements specific to each discourse are highlighted.

#### 3.2.1 Neoliberal discourse

A neoliberal discourse refers to a requirement for service users to comply with certain predetermined structures, such as standard procedures for evidence-based assessment and subsequent diagnosis and treatment. Here, health care services are governed by principles of efficiency and cost-benefit, which require fixed appointments of limited time. The participants talked about the fixed appointments in relation to the fact that they did not always need to talk at the scheduled times, and about the extensive forms with many repetitive questions, which prevented them from talking about what they needed to discuss, as in this example:

“So first I went to CAMHS and then I had one fixed appointment a week to meet them, and I was like, well, for me it didn’t work out very well, I’d been to CAMHS before, but it didn’t work properly because you have to complete those big forms before you can actually start talking. And what’s more, there are like fixed times, so that means maybe I don’t always need to talk at that time, it’s more necessary for me to talk when I need to talk (P9).”

This quotation illustrates how CAMHS discursive practice is shaped by a neoliberal discourse, which is reflected in how the participants experience of CAMHS is articulated, but where the participant suggests a preference for an alternative discourse that is more user-oriented and flexible.

This discourse and discursive practice also manifests in the way the participants in several ways discussed how busy the staff were and the efficiency of CAMHS. For example, they said that the staff did not have time to meet them outside the office, they could cancel the appointment when you were in the waiting room because another person needed it more, were late for appointments and could never continue beyond the scheduled time. The participants talked about how CAMHS was mechanical and strict and said that the staff were present in a very efficient way, as one said: ‘It is much easier for them (Youth Flexible ACT) to be present if something is needed not that they’re not present at CAMHS, but it’s in a completely different, very efficient way (P10).’.

When the participants compared treatment in Youth Flexible ACT with treatment in CAMHS, they highlighted that the former was more relaxed, less serious and not so strict. CAMHS was very strict in terms of flexibility of time and arena flexibility. One quotation comparing the two services shows how the inability of CAMHS to provide good treatment was explained and excused by the participant because CAMHS was seen as a system with many patients and a heavy workload: ‘I just wanted to say that it [Youth Flexible ACT] is better than the way CAMHS often is, but that doesn’t necessarily mean it is CAMHS’ problem. They have a lot of patients and a lot to do; it’s kind of a different system (P10).’.

A further aspect of a neoliberal discourse is expressed when the users are seen as consumers who are meant to act rationally and be responsible for utilising the services offered. For example, the participants expressed that they had difficulty keeping appointments, and said that in the CAMHS system the patients who didn’t come to appointments might not be allowed to continue. In the neoliberal mindset, it was the patient’s responsibility to be suitably engaged in treatment to continue. One example was a young person with a severe disability who had been in treatment for six months. He said that the staff had told him that he had not done enough and that he had to do something himself or they would terminate his treatment:

“I think I had been going there for a little over half a year, and there wasn’t much happening. Then they just said that now nothing’s happened or you’re not doing enough, so either you contact us in a month and tell us what you’ve done or we’ll leave it at that (P11).”

The participants mainly described a rigid system where a neoliberal discourse provided a framework that enacted the kind of flexible practices that these young people needed.

#### 3.2.2 Paternalistic discourse

A paternalistic discourse and discursive practice were also identified from the participants’ articulation about their treatment in CAMHS. This type of discourse provides guidance for what hierarchical decision-making structures to apply; for example, the perspectives and terminology of the staff determine the treatment. The system and staff make the decisions, while young patients have limited involvement in decision-making processes that affect their treatment. Several participants expressed having no say in their treatment, not being listened to and not being invited to cooperate with the staff. Examples of this were: ‘But they [CAMHS] don’t listen to what I want help with, they just do what they think, you know, but I feel that here in Youth Flexible ACT you get help with what you need help with (P8)’ and ‘the CAMHS people only cooperate with themselves (P12).’ This may reflect how the staff’s institutional objectives are prioritised over the participants’ individual goals and preferences. One example was a girl who had to comply with the procedure of having a crisis plan even though she clearly stated that it was against her wishes. The crisis plan was nevertheless drawn up, but without the girl being involved:

“I’m really against it [having a crisis plan] and he [Youth Flexible ACT staff member] respected that, but they didn’t respect it at CAMHS, where I had to have a crisis plan. Not that I was involved in making it, but my mother had to be involved in making it, because I was so much against a crisis plan, it just doesn’t work (P4).”

The participants’ statements illustrated power asymmetry in the relationship between the staff and themselves, where the participants had limited involvement, and the system and its staff set the agenda and were the experts with decision-making power. Another example of this paternalistic governance is how CAMHS decided that a young client could no longer receive treatment; CAMHS alone makes such decisions without clients being involved or prepared: ‘I’m always a bit worried that they (Youth FACT) will suddenly say I don’t need to come any longer, because that’s what happened at CAMHS and I didn’t handle it very well (P5).’.

Paternalism can also manifest itself as stigma. This can take the form of staff or others talking about patients in ways that reduce them to their disorder, or using a diagnostic category to describe them or in terms of a normal/abnormal dichotomy. This quotation from one of the participants included elements of paternalism in the form of stigma:

“So, like, when I was at CAMHS, you know, sitting there in the waiting room and I’m there, well, I just feel like more psychotic, like I’m not normal, you see? I feel very different from other people, and I know that going to CAMHS is a very normal thing, but I don’t feel it’s very normalised. Like once when I was going to CAMHS, I didn’t tell my friends ‘I’m going to CAMHS’, I just said ‘I’m leaving now, I have to go, I’ve got an appointment (P9).”

This participant talked about the distinction between normal and abnormal, where going to CAMHS made her feel different from others and place herself in the medical category of ‘psychotic’, while she kept her CAMHS appointment secret from her friends.

#### 3.2.3 Biomedical discourse

We also identified a prominent biomedical discourse and discursive practice, where the participants talked about diagnoses, medication, assessment and a focus on problems. They had to talk about their problems in CAMHS; they could not talk about what they wanted, such as their interests and ‘everything’. They also said that the problems had to be removed quickly with the easiest solution; one participant talked about the focus on problems by comparing it to Youth Flexible ACT: ‘We can talk about anything at all [in Youth Flexible ACT], you know, and I don’t even have to talk about my problems every time. So for example, I’ve talked about piercings, like I want to get new piercings, but at CAMHS I had to talk about my problems (P4).’.

Another topic was diagnosis. Participants found it helpful to know that they had a diagnosis, but they also felt that there was less focus on illness and more flexibility in Youth Flexible ACT than in CAMHS, where they were given a diagnosis; they viewed CAMHS as a place where the staff are trained to diagnose people: ‘[…] I don’t know if Youth Flexible ACT could have given me those diagnoses, because CAMHS is sort of trained to diagnose you with that. But I like the way Youth Flexible ACT is more flexible (P10)’.

Another way of talking about CAMHS in terms of a biomedical discourse was when one participant said that it was important for CAMHS to find a box to put people in, people should be categorised and then given medicine for the good of society. The participant’s quotation also referred to a paternalistic discourse whereby the treatment was meant to solve the problem for the good of society, not for the individual. This can be seen in the quotation, in addition to some overlapping between the discourses on treatment in CAMHS:

“[…]like they really want to find some kind of box they can put you in so they can give you medicine so you can be less of yourself, if you know what I mean. And they’re like, they’re not that interested in solving the problem, like what’s best for you, no, they want to solve the problems in the best way for society or maybe the people around you, you know (P1).”

Being ‘put in a box’ can be seen as a metaphor for categorisation and systematisation; young patients are reduced to diagnoses. The participant also explicitly stated that this was a solution that did not help them, but that by becoming less of themselves, they would be less of a burden on society or people around them.

### 3.3 Social practice

The third dimension of Fairclough’s framework is social practice. Analysis of social practice sheds light on the broader non-discursive social, cultural and institutional context in which the text and the discursive practice are shaped by and help to shape. The economic and institutional conditions that frame the discursive practice are particularly important in CDA (35) and will therefore be discussed here. In addition, the discourses will be discussed in the light of Norwegian health policy, including national guidelines and legislation.

The findings suggest that a neoliberal discourse is the prevailing framework in mental health care, where the system is governed by efficiency and cost-benefit and where individual patients are expected to take responsibility for using the treatment they are offered. Previous studies on service user involvement in mental health care align with our study in finding that a neoliberal discourse was prevalent in mental health care services (see e.g (41, 64). This discourse reflects a societal shift towards individualisation, which for children and young people and their parents in mental health care means that they are expected to act responsibly, be active and become as independent as possible of professionals. This discursive practice can be seen as a reflection of the broader social practice that Fairclough calls the ‘marketisation of discourses’, where a market discourse is seen to colonise discursive practices in public institutions (56). Since the 2000s, mental health care has similarly been subjected to reforms to improve efficiency, based on new public management and evidence-based knowledge (42). This economic form of governance focuses on quantity with measurable and standardised processes and accompanying documentation of compliance with legal requirements, along with the use of evidence-based and standardised methods. Frameworks of this kind lay down guidelines for the type of treatment to be provided and directly interfere with clinical practices and professional autonomy (64, 65). A shift towards neoliberalism in mental health policy may place the responsibility for problems increasingly on the individual and the family, and less on socio-economic factors (65, 66). Another possible consequence of such social practice is that relational and time-consuming care often does not ‘pay off’ in terms of the rationale of conventional health care services.

Service user participation in treatment is stipulated in legislation and is a key aspect of national guidelines for CAMHS. This right to participate is part of a system with paternalistic roots, where staff are professionals with decision-making power and where these young people are sometimes described as doubly vulnerable due to their age and their severe mental health problems. This may lead to them being seen as less competent to participate. A hierarchical structure and a biomedical model have also been dominant in traditional mental health care, which can further limit real participation for this group.

The biomedical discourse can be seen as being closely linked to the institutional framework, which is organised according to medical principles, evidence-based and standardised treatment, and documentation requirements. An unfortunate consequence can be that complex problems are adapted to categories, which can make young people feel that their complex and multifaceted difficulties are reduced to diagnoses and individual problems, in order to fit into the framework of the system.

These institutional and economic frameworks provide a supportive context for neoliberal and biomedical discourses, which normalise understandings of mental health care as efficient, standardised and diagnosis-driven. The young people’s ways of talking about CAMHS can thus be seen as part of a wider discursive practice that both reflects and reinforces these organisational priorities. At the same time, when participants draw on more flexible, relational and less illness-oriented ways of talking about Youth Flexible ACT, they introduce discursive elements that can be seen as challenging aspects of the prevailing social practice and as pointing towards possible alternative ways of conceptualising and delivering mental health care for young people.

## 4 Discussion

The participants in this study were young people with severe mental health problems and complex needs who were receiving treatment from Youth Flexible ACT teams and had experience with treatment in CAMHS. This provided them with a basis for comparing the different services, and the results show that many of the statements about CAMHS were made in explicit comparisons with Youth Flexible ACT. The young people had predominantly negative views of their treatment in CAMHS, although a few said it was acceptable. Using an analysis inspired by Fairclough’s critical discourse framework, we identified three discourse patterns: biomedical, paternalistic and neoliberal, in interview transcripts where participants articulated their past experiences with CAMHS. This framework allowed us to view the findings within a broader social and cultural context, providing insights into how the language used about CAMHS and the prevailing discourses painted a picture of a service that did not fulfil these young people’s wishes and needs.

### 4.1 Neoliberal, paternalistic and biomedical discourses in comparison with research and guidelines for CAMHS

The results of our analysis indicated that a *neoliberal discourse* prevailed, where the participants described a rigid system that lacked flexibility, with predetermined structures that governed the support they received. The participants consistently talked about time pressure in CAMHS, for example, with appointments cancelled, not being given appointments when needed, and being excluded if they were too well, too ill, or showed no progress. The participants also described a *paternalistic discourse* with little autonomy, primarily governed by the CAMHS system rather than the staff. They spoke of themselves as passive recipients instead of active participants. A biomedical discourse also dominated participants’ talk about CAMHS. They were assessed with forms, given diagnoses and medications, and expected to discuss and get rid of their problems, ideally quickly and for the benefit of society. This indicates how their challenges were to be understood, excluding other ways of discussing, understanding and treating mental health problems.

The findings partly concur with other studies on young people’s experiences of CAMHS, which show that young people described receiving good care and support, although many, like our participants, reported not being listened to or involved and experiencing discontinuity (24–26). These findings contrast with what young people say they want from CAMHS, which was nevertheless provided by Youth Flexible ACT, according to the participants. They reported collaborative, trust-based and friendship-like relationships with staff, and an accessible and flexible service (33). To our knowledge, no previous studies have used discourse analysis to investigate young people’s experiences of treatment in CAMHS. However, our findings are consistent with discourse analysis studies on user involvement and recovery in adult mental health services, where the same discourses were found to be dominant (41, 61, 66).

Despite national guidelines and frameworks for child and adolescent mental health services (19, 20) advocating service user involvement, personalised treatment and biopsychosocial models, in line with young people’s preferences, the results of this study indicate that CAMHS continues to practice a model largely based on a biomedical perspective, dominated by paternalistic and neoliberal discourses. This persists even though biomedical practices in psychiatry are often controversial or viewed as deficient (67, 68). This is further supported by the latest WHO’s Guidance saying: ‘While diagnosis, symptom reduction, and medical treatment are beneficial for some individuals in certain situations, a more comprehensive approach is needed: one that addresses the root causes of distress, often rooted in social and structural factors’ (47, p. 64)

### 4.2 Recovery, self-understanding and discourses

The young people’s use of language to describe treatment in CAMHS appears to suggest an object-based ontology, where biomedical language focuses on illness and problems, rather than a subject-based ontology aimed at improving health. Our transitivity analysis indicates how the participants’ use of passive language positions them in a passive role as recipients of treatment determined by professionals or the health care system. The young people’s passive language foregrounds what is done to them, while backgrounding who acts and why. In addition, their frequent reference to an anonymous ‘they’ who ‘can give you medicine so you can be less of yourself’ constructs CAMHS as an impersonal system rather than a set of identifiable relationships. These grammatical choices and pronoun patterns contribute to representing the young people as objects of treatment. This way of talking about CAMHS is divergent from a person-centred and recovery-oriented perspective, where involvement, personal resources, and the person’s thoughts about what is meaningful are central aspects (48, 49). At the level of social practice, this object-based ontology resonates with institutional and organisational conditions in CAMHS that prioritise diagnostic categorisation, standardised treatment pathways and efficiency, and thus reinforces biomedical, paternalistic and neoliberal ways of organising care. Experiences of CAMHS thus contrast with those of Youth Flexible ACT, where the participants described their interests and involvement as vital parts of the treatment (33), unlike the focus on problems and illness in CAMHS. When young people describe their mental health as an illness and their behaviour as an individual challenge, it diverts attention from socio-economic context, such as family poverty, disadvantaged housing and neighbourhoods (69). The tendency to psychologise social issues is not new (70). Treatments focusing primarily on the individual and enhancing autonomy contrast with holistic approaches, which emphasise systemic levels such as school, family and community. Holistic treatment at the systemic level has shown a greater positive impact on the mental health of vulnerable young people than changes the individual can make, such as altering cognition and behaviour (71).

A review of young people’s recovery processes found that they differed from adults by involving a more uncertain and fluctuating view of recovery, especially regarding recovery goals, which could be polarised. Their recovery processes are also often marked by a desire for diagnosis and symptom reduction (52). Young people’s views on recovery may thus align to some extent with the biomedical model, but from a discursive perspective, this may reflect the dominant cultural understanding where the biomedical model holds a hegemonic position, with a focus on diagnosis and symptoms being taken for granted as mitigating mental health problems. Being placed in a disease category like a ‘box’, as expressed by one of the participants, without being a participating agent, can negatively affect the development of young people’s self-concept, defined as an individual’s set of beliefs about themselves (72). Studies exploring young people’s understanding of diagnosis find positive functions, such as increased self-understanding, legitimisation of problems, and greater access to resources and treatment. Conversely, diagnosis can negatively impact self-concept by reducing self-worth through attributing negative traits to the person (73). Similar to our findings, others have found that receiving a diagnosis and encountering an authoritative attitude in health care professionals may feel judgemental and trigger resistance, thus becoming a barrier to participation in treatment (74). A study on young people’s experiences of being diagnosed with ADHD found a pattern where children from middle-class families generally had a more reflective approach to the diagnosis than those from working-class families. Working-class children identified more with the diagnosis, often viewing it as something negative about themselves (75). Findings indicate the importance of understanding the influence of a young person’s environment, as those with fewer socio-economic resources may be more vulnerable to a biomedical discourse and a diagnostic practice. Thus, the biomedical model need not be seen as conflicting with other perspectives; instead, helpful elements from multiple discourses can be combined to enhance one another and promote more comprehensive service provision. Perkins et al. (76), propose a comprehensive model that informs diagnostic practices in line with recovery-oriented principles, emphasising the importance of conducting diagnoses within a holistic and contextual framework.

Recovery can challenge paternalistic, neoliberal and biomedical dominance by focusing on autonomy, strengths and social and structural understandings of mental health issues, but it has also been criticised for evolving into ‘neorecovery’ (77). Critics view recovery as part of individualistic discourses such as ‘self-management’ and ‘person-centredness’, which, like neoliberalism, aim for independence while ignoring social structures and cultural context (78). Such practices may overlook ecological perspectives such as poverty and social justice as contributors to mental health problems (37). Like Jørgensen and Philips (40), we question whether a market-oriented health care model based on self-care is at all meaningful for vulnerable groups.

### 4.3 Frameworks and values in mental health care

The participants’ negative comments about CAMHS were striking. The analysis of truth modality suggested that their statements about CAMHS expressed a high degree of truth certainty, e.g. that CAMHS was of little help to them. The framework of traditional mental health services/CAMHS may be less suitable for children and adolescents with severe mental health problems and complex needs for several reasons. It is not designed to accommodate young people with trauma and relational distress/attachment disorders and the complex, multifaceted challenges of many in this target group (79, 80), which often require care and support over several years rather than for a limited period. Instead of flexible and long-term support, they are offered sessions based on a medical assumption of a necessary ‘treatment dosage’, which might be 50 minutes every second week. Young people are sometimes viewed by professionals and those around them as unmotivated or incapable of engaging in treatment instead of professionals considering whether the treatment is suited to their needs (81). The structure of conventional mental health care services risks reproducing inequalities and uneven power relations for this group, as the young people most engaged with the services tend to be those with strong social skills (82). A study also found that adolescents dissatisfied with the services they received in CAMHS struggled in many areas of life as young adults, unlike those who were more satisfied (83).

The field of mental health care has been criticised for having become a professional practice with a specific form, where the goal is to promote service users’ capacity for self-management and life coping skills (37, 84). The young people in this study had difficulty in utilising the treatment offered by CAMHS, attending appointments at specific times and being ready to talk during scheduled sessions. To be unable to benefit from the treatment offered can be yet another experience of failure, and loss of hope (85). A neoliberal discourse with efficiency and independence as goals may also be problematic for young people who are severely ill and struggle with painful and distressing symptoms, since support and care are then no longer objectives in themselves. It is difficult to encourage this group to engage in mental health care and treatment (18), and therapy resistance can occur as a result of dropout (86). It is therefore crucial that they receive treatment tailored to their particular difficulties when they enter care; key aspects here are perceived fair treatment, being heard and maintaining hope. Incorrect or inadequate treatment over time can be harmful, eroding hope for recovery and making it difficult to trust the support system again (87). It is important to discuss whether these young people should have conventional treatment, and for how long, before they receive better tailored care and support, such as Youth Flexible ACT, to individualise treatment as much as possible.

### 4.4 Implications

Our findings show that none of the identified discourses enable genuine participation or an active role for young people, revealing a persistent power imbalance in CAMHS. Aligned with Fairclough’s critical discourse analysis, this requires exposing how discursive and institutional practices sustain inequality and promoting approaches that redistribute power. We highlight some concrete practices that support more equal participation, such as: Involving young people directly in the development of their own treatment plans, thereby giving them influence over decisions that affect their lives; creating structured opportunities for young people to articulate their perspectives, for example, by using youth feedback tools; and using collaborative decision-making approaches that shift the practitioner’s role from an authoritative expert to a facilitator of dialogue.

The study also points to the importance of a broader, more holistic orientation that emphasises social factors, inclusion, and young people’s strengths. Framing young people’s challenges within an ecological context and emphasising the importance of various social factors and the interaction between individuals and their environment can enhance their understanding of being part of a larger context. Changes in language, values, and service culture, elements that can shift more rapidly than structural conditions, may reduce stigma, strengthen agency, and support recovery.

The findings further highlight the need for more differentiated service pathways. Young people should not be required to go through CAMHS before accessing specialised or better−suited support and must be able to transition quickly when needed. However, CAMHS operates within economic, legal, and administrative structures that often reflect neoliberal rationalities, making the implications relevant beyond the immediate study context.

### 4.5 Limitations and strengths of the study

This study has both strengths and limitations. All participants had experience with Youth Flexible ACT, which likely shaped how they perceived and articulated their earlier contact with CAMHS. Their perspectives are therefore situated within a specific service context. At the same time, ‘ experience with two services may have enabled more nuanced reflections on mental health care. The use of participant quotations strengthens transparency and credibility by grounding the analysis in empirical material.

A further strength is that the discursive patterns analysed through critical discourse analysis (CDA) emerged inductively from participants’ accounts rather than being predefined in the interview guide, potentially enhancing ecological validity. However, as the interviews were not specifically designed to explore institutional discourse and power relations, some discursive dimensions may not have been fully captured. The analysis should therefore be understood as a theoretically informed interpretation of naturally occurring interview data.

The study addresses an underexplored area: young people with severe mental health problems and their experiences with CAMHS. The use of CDA, which is relatively uncommon in this field, enables analysis beyond the individual level by linking personal accounts to broader institutional and societal discourses. This approach highlights how language and power shape service experiences, and can make visible complexities that can be overlooked in other methodological approaches.

Nonetheless, CDA has recognised limitations, including challenges in distinguishing discursive from non-discursive practices and in determining whether language reflects broader discourses or individual meaning-making. As an interpretive methodology, the analysis is shaped by the researchers’ theoretical positioning and analytical choices. We sought to enhance rigour through reflexivity, transparency, and close connection between interpretations and participants’ accounts. The authors reflected on our experiences in the clinical and research field, and on the discourses in which we are embedded. As a research group we have several years of research experience in mental health services, primarily for adults. We have clinical experience in Educational and Psychological Counselling Service and mental health services for children, young people and adults. Two authors are clinical psychologists, two are sociologists and one have cand.polit in pedagogical psychology. Four have Ph.d.s, two have professor position and one is a Ph.d. candidate.

Finally, convenience sampling limits statistical generalisability. However, participants represented a diverse group with substantial psychological and social challenges. Rather than aiming for generalisation, the study seeks to provide conceptual insights that may be transferable to similar contexts.

### 4.6 Conclusion

This study offers insight into how young people with severe mental health problems and complex needs articulate their treatment in CAMHS, and contrast it to treatment in Youth Flexible ACT. Through Fairclough’s critical discourse analysis, we identified three dominant and overlapping discourses that structured how participants made sense of their experiences. Together, these discourses positioned young people primarily as objects of diagnosis, regulation and efficiency-driven treatment, rather than as active subjects in their own recovery processes. The findings reveal a gap between the envisioned organisation of mental health services, as described in guidelines and by young people themselves. While these sources emphasise service user involvement and biopsychosocial models, participants in this study described a different reality. Importantly, when contrasting CAMHS with Youth Flexible ACT, participants drew on more relational and participatory language, suggesting that alternative discursive and organisational arrangements are both imaginable and already emerging.

The findings highlight the need for service differentiation and faster access to tailored treatment to prevent these young people from being subjected to ineffective or harmful interventions before receiving appropriate help. In addition, the findings underscore the need for practices that support more equal participation in care.

The institutional and economic framework, shaped by neoliberal governance, sets conditions for what kind of treatment staff in CAMHS can provide to this group of young people. Our findings suggest that this framework is neither meaningful nor suitable for working with this group. By contrast, mental health services organised differently, such as Youth Flexible ACT, may represent a more relevant alternative, offering a coherent service framework aimed at facilitating holistic, integrated and recovery-oriented care.

Future research should build directly on these findings. First, studies are needed that examine how biomedical, paternalistic and neoliberal logics are enacted in everyday clinical encounters, including interactional analyses of professional–youth communication. Second, longitudinal research should explore how diagnostic and categorising practices influence young people’s self-understanding, identity development and recovery trajectories over time. Third, research should critically investigate how institutional and economic frameworks shape service practices and constrain relational work. Finally, comparative and participatory studies are needed to explore and evaluate alternative service models (like Youth Flexible ACT) that explicitly aim to strengthen agency, flexibility and social inclusion.

By situating young people’s accounts within broader discursive and structural conditions, this study underscores that improving services requires not only new interventions, but critical reflection on the language, power relations and organisational logics that shape mental health care.

## Data Availability

The datasets presented in this article are not readily available to protect confidentiality. Requests to access the datasets should be directed to marthe.johansen@inn.no.

## References

[1] Solmi M Radua J Olivola M Croce E Soardo L Salazar de Pablo G . Age at onset of mental disorders worldwide: Large-scale meta-analysis of 192 epidemiological studies. Mol Psychiatry. (2022) 27:281–95. doi: 10.1038/s41380-021-01161-7. PMID: 34079068 PMC8960395

[2] McGorry PD Mei C Chanen A Hodges C Alvarez‐Jimenez M Killackey E . Designing and scaling up integrated youth mental health care. World Psychiatry. (2022) 21:61–76. doi: 10.1002/wps.20938. PMID: 35015367 PMC8751571

[3] Radez J Reardon T Creswell C Lawrence PJ Evdoka-Burton G Waite P . Why do children and adolescents (not) seek and access professional help for their mental health problems? A systematic review of quantitative and qualitative studies. Eur Child Adolesc Psychiatry. (2021) 30:183–211. doi: 10.1007/s00787-019-01469-4. PMID: 31965309 PMC7932953

[4] Aguirre Velasco A Silva Santa Cruz I Billings J Jimenez M Rowe S . What are the barriers, facilitators and interventions targeting help-seeking behaviours for common mental health problems in adolescents? A systematic review. Focus. (2025) 23:98–118. doi: 10.1176/appi.focus.25023003. PMID: 39776457 PMC11701830

[5] Van den Steene H Van West D Glazemakers I . Towards a definition of multiple and complex needs in children and youth: Delphi study in Flanders and international survey. Scandinavian J Child Adolesc Psychiatry Psychol. (2019) 7:60. doi: 10.21307/sjcapp-2019-009. PMID: 33520769 PMC7709935

[6] Bansema C Vermeiren R De Soet R Van Ewijk H Nijland L Nooteboom L . A systematic review exploring characteristics of youth with severe and enduring mental health problems (Semhp). Eur Child Adolesc Psychiatry. (2024) 33:1313–25. doi: 10.1007/s00787-023-02216-6. PMID: 37093338 PMC11098915

[7] Gulliver A Griffiths KM Christensen H . Perceived barriers and facilitators to mental health help-seeking in young people: A systematic review. BMC Psychiatry. (2010) 10:1–9. doi: 10.1186/1471-244x-10-113. PMID: 21192795 PMC3022639

[8] Markoulakis R Cader H Chan S Kodeeswaran S Addison T Walsh C . Transitions in mental health and addiction care for youth and their families: A scoping review of needs, barriers, and facilitators. BMC Health Serv Res. (2023) 23:470. doi: 10.1186/s12913-023-09430-7. PMID: 37165343 PMC10171912

[9] Fusar-Poli P . Integrated mental health services for the developmental period (0 to 25 years): A critical review of the evidence. Front Psychiatry. (2019) 10:355. doi: 10.3389/fpsyt.2019.00355. PMID: 31231250 PMC6567858

[10] Settipani CA Hawke LD Cleverley K Chaim G Cheung A Mehra K . Key attributes of integrated community-based youth service hubs for mental health: A scoping review. Int J Ment Health Syst. (2019) 13:1–26. doi: 10.1186/s13033-019-0306-7. PMID: 31367230 PMC6651922

[11] Skokauskas N Fung D Flaherty LT Von Klitzing K Pūras D Servili C . Shaping the future of child and adolescent psychiatry. (2019) 13:1–7. doi: 10.1186/s13034-019-0279-y, PMID: 31007713 PMC6458731

[12] World Health Organization . Integrated care models: An overview (2016). Available online at: https://iris.who.int/bitstream/handle/10665/375502/WHO-EURO-2016-6525-46291-66959-eng.pdf?sequence=1 (Accessed February 2, 2025).

[13] Cohen M . A systemic approach to understanding mental health and services. Soc Sci Med. (2017) 191:1–8. doi: 10.1016/j.socscimed.2017.08.037. PMID: 28881215

[14] Nooteboom LA Mulder EA Kuiper CH Colins OF Vermeiren RR . Towards integrated youth care: A systematic review of facilitators and barriers for professionals. Administration Policy Ment Health Ment Health Serv Res. (2021) 48:88–105. doi: 10.1007/s10488-020-01049-8. PMID: 32424453 PMC7803720

[15] Melby L Ulset G Paulsen V Wågø S Høyland K Kaasbøll J . Nytt institusjonstilbud for unge med samtidig behov for omsorg og psykisk helsehjelp. sluttrapport. [New institutional services for youth with concurrent needs for care and mental health support, final report]. [SINTEF (2020). Available online at: https://hdl.handle.net/11250/2671744 (Accessed May 19, 2025).

[16] Barneombudet . Jeg skulle hatt Bup i en koffert. En psykisk helsetjeneste tilpasset barn og unges behov [I should have had Bup in a suitcase. A mental health service adapted to the needs of children and youth (2020). Available online at: https://www.barneombudet.no/uploads/documents/Publikasjoner/Fagrapporter/Jeg-skulle-hatt-BUP-i-en-koffert.pdf (Accessed May 25, 2025).

[17] Hansen ILS Jensen RS Fløtten T . Trouble in the interfaces. Coordinated effort directed at vulnerable children and young people. [FAFO (2020). Available online at: https://www.fafo.no/zoo-publikasjoner/fafo-rapporter/trobbel-i-grenseflatene (Accessed May 19, 2025).

[18] Woody C Baxter A Wright E Gossip K Leitch E Whiteford H . Review of services to inform clinical frameworks for adolescents and young adults with severe, persistent and complex mental illness. Clin Child Psychol Psychiatry. (2019) 24:503–28. doi: 10.1177/1359104519827631. PMID: 30818969

[19] Norsk barne- og ungdomspsykiatrisk forening . Faglig Veileder for Barne- Og Ungdomspsykiatri [Clinical Guideline for Child and Adolescent Psychiatry]. Oslo: Den norske legeforening (2016). Available online at: https://www.legeforeningen.no/contentassets/308e31a34fb84ab59b7fe2cd0159c476/bup-med-innholdsfortegnelse-nt010719.pdf (Accessed May 26, 2025).

[20] Helsedirektoratet . Veileder for poliklinikker i psykisk helsevern for barn og unge [Mental Health Services for Children and Adolescents - a guide for Outpatient Clinics]. Oslo: Helsedirektoratet (2008). Available online at: https://urn.nb.no/URN:NBN:no-nb_digibok_2014032606039 (Accessed May 25, 2025).

[21] Nord-Bade S . Model description youth flexible assertive community treatment (2022). Available online at: https://napha.no/multimedia/12118/FACT-ung-modellbeskrivelse (Accessed May 19, 2025).

[22] van Veldhuizen JR Bähler M . Flexible assertive community treatment (2013). Available online at: chrome-extension://efaidnbmnnnibpcajpcglclefindmkaj/https://ccaf.nl/wp-content/uploads//2015/06/FACT-Manual-ENGLISH-2013.pdf (Accessed February 3, 2025).

[23] Bähler M Delespaul P Kroon H van Vugt M Westen K . Workbook youth fact fidelity scale. The Netherlands: Groningen (2020). Available online at: https://ccaf.nl/wp-content/uploads/sites/2/2020/05/Youth-FACT-fidelity-scale-2020.pdf (Accessed February 2, 2025).

[24] Foster M Frith H John M . ‘I'm still su! c! dal when you're done with the paperwork’: An inductive framework thematic analysis of# camhs on tiktok. J Child Psychol Psychiatry. (2024) 65:1258–69. doi: 10.1111/jcpp.14002. PMID: 38724448

[25] Persson S Hagquist C Michelson D . Young voices in mental health care: Exploring children’s and adolescents’ service experiences and preferences. Clin Child Psychol Psychiatry. (2017) 22:140–51. doi: 10.1177/1359104516656722. PMID: 27368712

[26] Coyne I McNamara N Healy M Gower C Sarkar M McNicholas F . Adolescents' and parents' views of child and adolescent mental health services (CAMHS) in I reland. J Psychiatr Ment Health Nurs. (2015) 22:561–9. doi: 10.1111/jpm.12215. PMID: 25977175

[27] Plaistow J Masson K Koch D Wilson J Stark RM Jones PB . Young people's views of UK mental health services. Early Interv Psychiatry. (2014) 8:12–23. doi: 10.1111/eip.12060. PMID: 23773401

[28] Martin A DiGiovanni M Acquaye A Ponticiello M Chou DT Neto EA . Pathways and identity: Toward qualitative research careers in child and adolescent psychiatry. Child Adolesc Psychiatry Ment Health. (2024) 18:49. doi: 10.1186/s13034-024-00738-8. PMID: 38685108 PMC11059710

[29] De Soet R Nooteboom L Bansema C Van Ewijk H Nijland L Vermeiren R . How to meet the needs of youth with severe and enduring mental health problems: A qualitative study to barriers and facilitators in treatment. Children Youth Serv Rev. (2023) 155:107155. doi: 10.1016/j.childyouth.2023.107155. PMID: 41862359

[30] Green CA Wisdom JP Wolfe L Firemark A . Engaging youths with serious mental illnesses in treatment: Stars study consumer recommendations. Psychiatr Rehabil J. (2012) 35:360–8. doi: 10.1037/h0094494. PMID: 23116376 PMC3536447

[31] Davison J Zamperoni V Stain HJ . Vulnerable young people’s experiences of child and adolescent mental health services. Ment Health Rev J. (2017) 22:95–110. doi: 10.1108/mhrj-09-2016-0016. PMID: 35579975

[32] Kapur P Hayes D Waddingham R Hillman S Deighton J Midgley N . The experience of engaging with mental health services among young people who hear voices and their families: A mixed methods exploratory study. BMC Health Serv Res. (2014) 14:1–9. doi: 10.1186/s12913-014-0527-z. PMID: 25371020 PMC4256811

[33] Johansen M Stuen HK Brekke E Jensen CB Landheim A . A qualitative study of the experiences of young people with severe mental health problems and complex needs regarding youth flexible assertive community treatment. Front Psychiatry. (2024) 15:1478345. doi: 10.3389/fpsyt.2024.1478345. PMID: 39713766 PMC11662976

[34] Bevington D Fuggle P Fonagy P Target M Asen E . Innovations in practice: Adolescent mentalization‐based integrative therapy (Ambit)–a new integrated approach to working with the most hard to reach adolescents with severe complex mental health needs. Child Adolesc Ment Health. (2013) 18:46–51. doi: 10.1111/j.1475-3588.2012.00666.x. PMID: 32847259

[35] Jørgensen M Philips L . Diskursanalyse som teori og metode. 12. Cambridge: Roskilde Universitetsforlag (2021).

[36] Beedholm K Lomborg K Frederiksen K . Discourse analysis and the impact of the philosophy of e nlightenment in nursing research. Nurs Inq. (2014) 21:112–20. doi: 10.1111/nin.12034. PMID: 23679133

[37] Rose N . Our psychiatric future Vol. 1. Cambridge: Polity (2018).

[38] Accordini M Saita E Irtelli F Buratti M Savuto G . Stories of change: The text analysis of handovers in an Italian psychiatric residential care home. J Psychiatr Ment Health Nurs. (2017) 24:232–42. doi: 10.1111/jpm.12377. PMID: 28198578

[39] Hansen JO Huniche L Nielsen CT Petersen A . The politics of mental illness and involvement: A discourse analysis of Danish anti-stigma and social inclusion campaigns. Adv Appl Sociology. (2015) 5:273–85. doi: 10.4236/aasoci.2015.511026

[40] Jørgensen K Praestegaard J Holen M . The conditions of possibilities for recovery: A critical discourse analysis in a Danish psychiatric context. J Clin Nurs. (2020) 29:3012–24. doi: 10.1111/jocn.15311. PMID: 32353905

[41] Jørgensen K Rasmussen T Hansen M Andreasson K Karlsson B . User involvement in the handover between mental health hospitals and community mental health: A critical discourse analysis. Int J Environ Res Public Health. (2021) 18:3352. doi: 10.3390/ijerph18073352. PMID: 33805037 PMC8038082

[42] Ekeland T-J . Frå objekt til subjekt–og tilbake?-om tilhøvet mellom kunnskap, praksis og styring [from object to subject – and back? – on the relationship between knowledge, practice, and governance. Tidsskrift For psykisk helsearbeid. (2014) 11:211–20. doi: 10.18261/ISSN1504-3010-2014-03-03

[43] Deacon BJ . The biomedical model of mental disorder: A critical analysis of its validity, utility, and effects on psychotherapy research. Clin Psychol Rev. (2013) 33:846–61. doi: 10.1016/j.cpr.2012.09.007. PMID: 23664634

[44] Wampold BE . The great psychotherapy debate: Models, methods, and findings. New York: Routledge (2013).

[45] Engel GL . The need for a new medical model: A challenge for biomedicine. Science. (1977) 196:129–36. doi: 10.1126/science.847460. PMID: 847460

[46] Tripathi A Das A Kar SK . Biopsychosocial model in contemporary psychiatry: Current validity and future prospects. Indian J psychol Med. (2019) 41:582–5. doi: 10.4103/IJPSYM.IJPSYM_314_19. PMID: 31772447 PMC6875848

[47] World Health Organization . Guidance on mental health policy and strategic action plans. Module 4. Country case scenarios. Geneva: World Health Organization. (2025). Available online at: https://iris.who.int/bitstream/handle/10665/380468/9789240106857-eng.pdf (Accessed December 2, 2025).

[48] Ward D . ‘Recovery’: Does it fit for adolescent mental health? J Child Adolesc Ment Health. (2014) 26:83–90. doi: 10.2989/17280583.2013.877465. PMID: 25391573

[49] Le Boutillier C Leamy M Bird VJ Davidson L Williams J Slade M . What does recovery mean in practice? A qualitative analysis of international recovery-oriented practice guidance. Psychiatr Serv. (2011) 62:1470–6. doi: 10.1176/appi.ps.001312011. PMID: 22193795

[50] Naughton JNL Maybery D Sutton K . Review of child and adolescent mental health recovery literature: Concordance and contention. J Psychosocial Rehabil Ment Health. (2018) 5:151–8. doi: 10.1007/s40737-018-0119-z. PMID: 41859209

[51] Rayner S Thielking M Lough R . A new paradigm of youth recovery: Implications for youth mental health service provision. Aust J Psychol. (2018) 70:330–40. doi: 10.1111/ajpy.12206. PMID: 41858021

[52] Moberg J Skogens L Schön UK . Review: Young people's recovery processes from mental health problems – a scoping review. Child Adolesc Ment Health. (2023) 28:393–407. doi: 10.1111/camh.12594. PMID: 35960215

[53] United Nations . Convention on the rights of the child. Article 12. New York: United Nations (1989). Available online at: https://www.ohchr.org/en/instruments-mechanisms/instruments/convention-rights-child (Accessed February 10, 2025).

[54] Gergen KJ . Invitation to social construction co-creating the future. Los Angeles: SAGE Publications (2022).

[55] Fairclough N . Analysing discourse textual analysis for social research. London: Routledge (2003).

[56] Fairclough N . Kritisk diskursanalyse en tekstsamling. København: Hans Reitzels Forlag (2008).

[57] Fairclough N . Discourse and text: Linguistic and intertextual analysis within discourse analysis. Discourse Soc. (1992) 3:193–217. doi: 10.1177/0957926592003002004. PMID: 41836481

[58] Fairclough N . Language and power. Harlow: Longman (2001).

[59] Obilor EI . Convenience and purposive sampling techniques: Are they the same. Int J Innovative Soc Sci Educ Res. (2023) 11:1–7.

[60] World Medical Association . Declaration of Helsinki: Ethical principles for medical research involving human subjects. Helsinki: World Medical Association (1964). doi: 10.1515/9783110208856.233, PMID:

[61] Jørgensen K Hansen MS Hansen M Karlsson B . Health professionals' perceptions of user involvement in a mental health centre: A critical discourse analysis. Int J Ment Health Nurs. (2024) 33:937–948. doi: 10.1111/inm.13296. PMID: 38251782

[62] Skrede J . Kritisk diskursanalyse[Critical discourse analysis]. Oslo: Cappelen Damm Akademisk (2017).

[63] Bryde Christensen AB Dyrloev K Hoej M Poulsen S Reinholt N Arnfred S . ‘The depressed’ and ‘people with anxiety’ therapists’ discursive representations of patients with depression and anxiety in Danish Psychiatry. Health. (2024) 28:390–411. doi: 10.1177/13634593231173802. PMID: 37191112

[64] Karlsson B . Markedsliberalistiske forskyvninger i det psykiske helsefeltet–om forholdet mellom politisk styring og faglig disiplinering [Neoliberal shifts in the mental health field – on the relationship between political governance and professional disciplining. Nordisk Tidsskrift For Helseforskning. (2015) 11:153–62. doi: 10.7557/14.3719. PMID: 41850708

[65] Teghtsoonian K . Depression and mental health in neoliberal times: A critical analysis of policy and discourse. Soc Sci Med. (2009) 69:28–35. doi: 10.1016/j.socscimed.2009.03.037. PMID: 19427730

[66] Joergensen K Praestegaard J . Patient participation as discursive practice—a critical discourse analysis of Danish mental healthcare. Nurs Inq. (2018) 25:e12218-n/a. doi: 10.1111/nin.12218. PMID: 28940834

[67] Whitaker R Cosgrove L . Psychiatry under the influence: institutional corruption, social injury, and prescriptions for reform. New York: Springer (2015).

[68] Johnstone L Boyle M . The power threat meaning framework: An alternative nondiagnostic conceptual system. J Humanistic Psychol. (2018) 65:0022167818793289. doi: 10.1177/0022167818793289. PMID: 41836481

[69] Kirkbride JB Anglin DM Colman I Dykxhoorn J Jones PB Patalay P . The social determinants of mental health and disorder: Evidence, prevention and recommendations. World Psychiatry. (2024) 23:58–90. doi: 10.1002/wps.21160. PMID: 38214615 PMC10786006

[70] Rose N . Powers of freedom: reframing political thought. Cambridge: Cambridge University Press (1999).

[71] Ungar M Ghazinour M Richter J . Annual research review: What is resilience within the social ecology of human development? J Child Psychol Psychiatry. (2013) 54:348–66. doi: 10.1111/jcpp.12025. PMID: 23215898

[72] Baumeister RF . The self in social psychology. Philadelphia: Psychology Press (1999).

[73] O'Connor C Kadianaki I Maunder K McNicholas F . How does psychiatric diagnosis affect young people's self-concept and social identity? A systematic review and synthesis of the qualitative literature. Soc Sci Med. (2018) 212:94–119. doi: 10.1016/j.socscimed.2018.07.011. PMID: 30029092

[74] Bjønness S Grønnestad T Storm M . I’m not a diagnosis: Adolescents’ perspectives on user participation and shared decision-making in mental healthcare. Scandinavian J Child Adolesc Psychiatry Psychol. (2020) 8:139. doi: 10.21307/sjcapp-2020-014. PMID: 33564630 PMC7863730

[75] Olsvold A . «I am an ordinary boy»: Children´s tales about ADHD. Tidsskrift For Norsk Psykologforening. (2014) 51:857–858.

[76] Perkins A Ridler J Browes D Peryer G Notley C Hackmann C . Experiencing mental health diagnosis: A systematic review of service user, clinician, and carer perspectives across clinical settings. Lancet Psychiatry. (2018) 5:747–64. doi: 10.1016/S2215-0366(18)30095-6. PMID: 29680468

[77] Cohen CI . Neorecovery: A critical analysis of the relationship between neoliberalism and the recovery movement. Community Ment Health J. (2025) 61:248–53. doi: 10.1007/s10597-024-01275-6. PMID: 38874799

[78] Karadzhov D . Personal recovery and socio-structural disadvantage: A critical conceptual review. Health (London). (2023) 27:201–25. doi: 10.1177/13634593211014250. PMID: 33962518 PMC9923205

[79] MacBeth A Gumley A Schwannauer M Fisher R . Attachment states of mind, mentalization, and their correlates in a first‐episode psychosis sample. Psychol Psychotherapy: Theory Res Pract. (2011) 84:42–57. doi: 10.1348/147608310X530246. PMID: 22903830

[80] Broersen M Frieswijk N Kroon H Vermulst AA Creemers DHM . Young patients with persistent and complex care needs require an integrated care approach: Baseline findings from the multicenter youth flexible act study. Front Psychiatry. (2020) 11:609120. doi: 10.3389/fpsyt.2020.609120. PMID: 33324268 PMC7724087

[81] Barca TB Moltu C Veseth M Fjellheim G Stige SH . The nature of youth in the eyes of mental-health care workers: Therapists' conceptualization of adolescents coming to therapy at others' initiative. Int J Ment Health Syst. (2020) 14:31. doi: 10.1186/s13033-020-00363-w. PMID: 32391078 PMC7201627

[82] Head BW . Why not ask them? Mapping and promoting youth participation. Children Youth Serv Rev. (2011) 33:541–7. doi: 10.1016/j.childyouth.2010.05.015. PMID: 41862359

[83] Westbye OS Lydersen S Schei J . User satisfaction in former patients in child and adolescent psychiatry. Norwegian J Clin Nurs. (2022) 17:88502. doi: 10.4220/Sykepleienf.2022.88502. PMID: 41213920

[84] Voronka J . The politics of 'people with lived experience' experiential authority and the risks of strategic essentialism. Philosophy Psychiatry Psychol. (2016) 23:189–201. doi: 10.1353/ppp.2016.0017. PMID: 34409987

[85] de Soet R Vermeiren R Bansema CH van Ewijk H Nijland L Nooteboom LA . Drop-out and ineffective treatment in youth with severe and enduring mental health problems: A systematic review. Eur Child Adolesc Psychiatry. (2024) 33:3305–19. doi: 10.1007/s00787-023-02182-z. PMID: 36882638 PMC11564352

[86] Herpers PCM Neumann JEC Staal WG . Treatment refractory internalizing behaviour across disorders: An aetiological model for severe emotion dysregulation in adolescence. Child Psychiatry Hum Dev. (2021) 52:515–32. doi: 10.1007/s10578-020-01036-y. PMID: 32748274 PMC8113221

[87] Lundkvist‐Houndoumadi I Thastum M . Anxious children and adolescents non‐responding to CBT: Clinical predictors and families' experiences of therapy. Clin Psychol Psychother. (2017) 24:82–93. doi: 10.1002/cpp.1982. PMID: 26514088

