# Supplementary material for: ‘They really want to find some kind of box they can put you in’: how young people with severe mental health problems and complex needs talk about their past treatment in traditional child and adolescent mental health services - a critical discourse analysis
Source: Front Psychiatry. 2026 Apr 14;17:1759231. doi: 10.3389/fpsyt.2026.1759231 (PMC13122592; doi:10.3389/fpsyt.2026.1759231)
Supplement: Supplementary file 1 [file Table1.docx]

Supplementary Material

Table 1. (Appendix 1).

Overview of word-level analysis, semantic categorisation, and identified discourses

| **Discourses** | **Adjective** | **Noun** | **Pronoun** | **Verb** |
| --- | --- | --- | --- | --- |
| **Neoliberal** | Fixed  Easiest  Mechanical  Strict | Appointments (fixed)  Solution (easiest)  Forms  Lack of time  An efficient way | They  You | Leave (staff leave) |
| **Paternalistic** | Rigid  Inflexible  Strict | Offices  System  Forms  Diagnoses | They  I | Do not listen  Do not help  Do not cooperate |
| **Biomedical** | Big, long  Many  Repetitive  Mechanical  Bad  Formal  Poor/Pitiable  Different  Not normal  Psychotic | Diagnoses (Get  Received)  Psychosis  Problems  Assessment -form (Big, long)  Emergency -phone  Offices  ADHD medication  Questions (Many,  Repetitive)  The problems  Box  Effective way  Psychologist  Waiting room | They  I | Get  Received  Did not work |

**
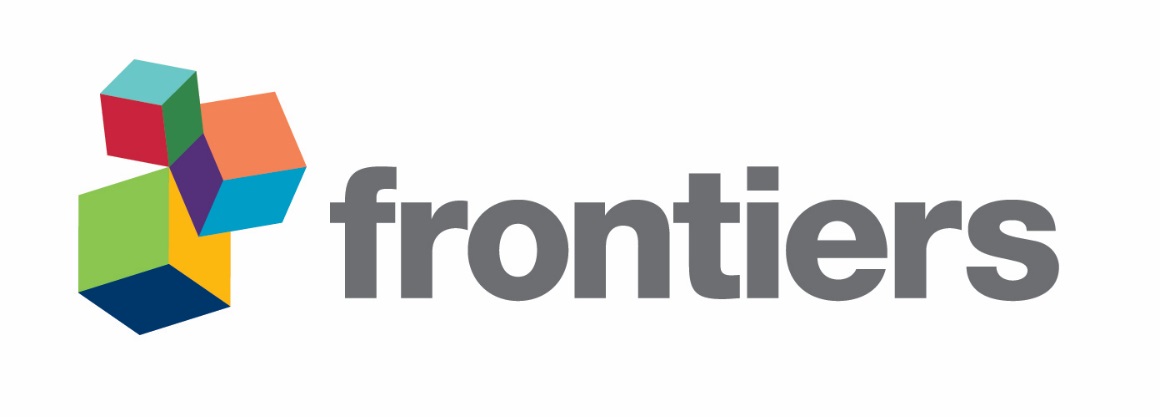
**
